# Complex Dependence of *Escherichia coli*-based Cell-Free Expression on Sonication Energy During Lysis

**DOI:** 10.1021/acssynbio.3c00312

**Published:** 2023-09-19

**Authors:** Fernanda Piorino, Mark P. Styczynski

**Affiliations:** School of Chemical & Biomolecular Engineering, Georgia Institute of Technology, 311 Ferst Drive NW, Atlanta, Georgia 30332-0100, United States

**Keywords:** cell-free expression systems, lysis, sonication, protein expression, variability

## Abstract

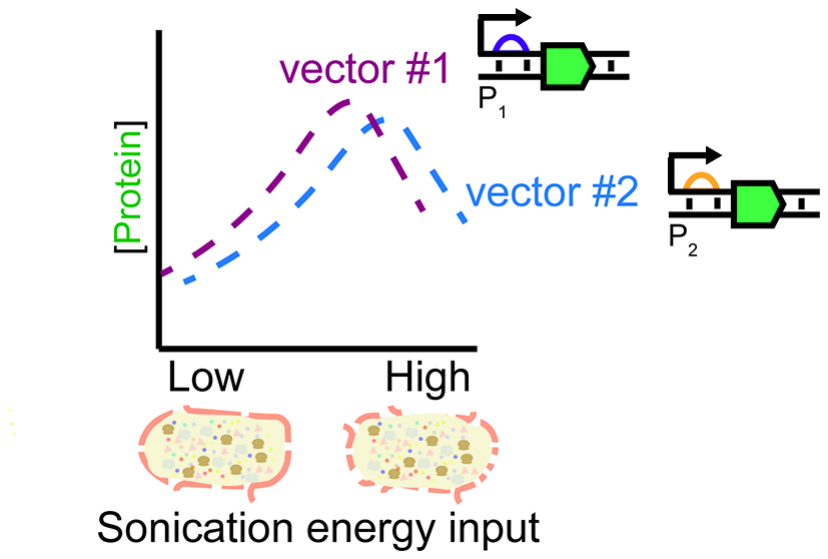

Cell lysis—by sonication or bead beating, for example—is a key step in preparing extracts for cell-free expression systems. To create high protein-production capacity extracts, standard practice is to lyse cells sufficiently to thoroughly disrupt the membrane and thus extract expression machinery but without degrading that machinery. Here, we investigate the impact of different sonication energy inputs on the protein-production capacity of *Escherichia coli* extracts. While the existence of operator-specific optimal sonication energy inputs is widely known, our findings show that the sonication energy input that yields maximal protein output from a given expression template may depend on plasmid concentration, transcriptional and translational features (e.g., promoter), and other expression vector components (e.g., origin of replication). These results indicate that sonication protocols cannot be standardized to a single optimum, suggest strategies for improving protein yields, and more broadly highlight the need for better metrics and protocols for characterizing cell extracts.

## Introduction

Although lysate-based cell-free expression (CFE) systems have become a powerful tool in a variety of synthetic biology applications, our understanding of these systems remains incomplete and the crude extract’s ultimate productivity is still plagued by unexplained variability.^1−3^ The lysate preparation protocol is complex, and multiple steps within it are potential sources of variation, including cell harvest and lysis. To improve reproducibility, comprehensive protocols that seek to streamline and standardize lysate preparation across the scientific community have been developed;^4−6^ these efforts have focused on *Escherichia coli*-based lysates, which are the most widely used CFE systems. While these protocols provide useful guidelines, their analysis often focuses solely on lysate characterization with model plasmids used to produce high protein titers, usually via a promoter taken from the T7 phage.

Notably, the extent and method of cell lysis can have a substantial impact on the extract’s ultimate protein-production capacity, making it a difficult step to standardize. Different lysis methods can be used, including sonication,^4,7^ bead vortex mixing,^6,7^ enzymatic lysis,^8^ and homogenization.^9^ Given its relatively low cost and reasonably wide availability, sonication has been widely used in laboratory settings.^7^ The extent of sonication can be controlled by, at fixed amplitude and burst time, monitoring the increase in energy input (e.g., in joules) while cycling between sonication bursts and cooling. The ideal energy input that results in maximal recombinant protein production can vary depending on the bacterial strain, the cell resuspension volume, the sonicator model, the immersion depth of the sonicator tip,^10^ and even the operator’s technique.^4^ However, once those variables are fixed, there seems to be a local optimum in energy that balances efficient membrane disruption with degradation of transcriptional and translational machineries, potentially due to heat shock. Although there are general guidelines for sonication energies with associated empirical correlations,^4^ each operator usually resorts to determining their optimal energy input experimentally.

Our group has previously studied lysates prepared at different sonication energy inputs using metabolomics. These lysates were found to have total protein concentration proportional to the sonication energy input. When normalized to have the same total protein concentration as the lysate prepared at the lowest sonication energy, these lysates still produced different titers of a reporter protein from a native *E. coli* promoter,^11^ indicating that their protein-production capacities were not strictly dictated by their transcriptional and translational machinery concentrations. In addition, sonication energy input was found to affect endogenous lysate metabolism: lysates prepared using different sonication energies had different metabolite profiles, which may be related to the differences in their productivities.^11^

Here, we advance the assessment of the impact of different sonication energy inputs on the protein-production capacity of *E. coli* BL21 (DE3) lysates. Specifically, we characterize this impact on superfolder GFP (sfGFP) expression from different promoters (from T7 phage and from *E. coli*), ribosomal binding sites (RBSs), and stability hairpins in the 5′ untranslated region (UTR) and in different plasmid vectors. We show that the optimal sonication energy input may not be a generalizable parameter; instead, constructs containing different transcriptional, translational, and other elements exhibit maximal protein titers in lysates prepared with different sonication energies.

## Results and Discussion

**Figure 1:**
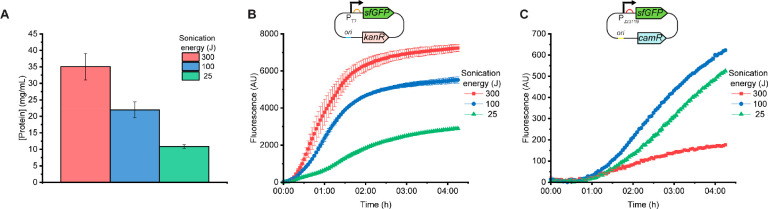
Characterization of three lysates prepared using different sonication energy inputs. (A) Total protein content as assessed via a Bradford assay. Protein content decreases with energy input, and the 300 J lysate has protein content similar to highly productive extracts reported in literature. (B) sfGFP expression from a T7 promoter. Fluorescence increases with increased energy input. The plasmid, pP_T7_-sfGFP, uses a high-copy vector that includes the 5′ UTR and RBS from gene 10 of T7 phage and has a kanamycin resistance gene (*kanR*). (C) sfGFP expression from P_J23119_, a strong σ^70^ promoter. Fluorescence is highest at 100 J, followed by 25 and 300 J. The plasmid, pP_J23119_-sfGFP, uses a low-copy vector that includes a strong RBS and has a chloramphenicol resistance gene (*camR*). In panels B and C, plasmid was added at 10 nM and data were collected during incubation at 37 °C. Error bars indicate the standard deviation of three technical replicates of a representative batch.

For the authors, a sonication energy input of approximately 300 J for a 1 mL BL21 (DE3) cell suspension yields strong recombinant protein expression from a T7 promoter (commonly used for high-titer CFE) and a total protein content in line with reports using standard protocols^6^ (Figure 1A). A sonication energy input higher than 300 J (350 J) yielded less sfGFP expression from a T7 promoter (Figure S1). An input higher than 350 J did not enable successful extract preparation, as the postsonication separation between cell pellet and supernatant was not clear.

We then prepared extracts lysed at our previously identified “ideal” energy input of 300 J as well as two lower sonication energies, 100 and 25 J. The 100 and 25 J lysates had lower total protein content (Figure 1A), likely due to incomplete lysis. We tested these extracts’ ability to produce sfGFP from two vectors based on plasmids we and other groups routinely use to characterize lysate preparations,^12^ achieve high-titer CFE,^13^ and implement cell-free applications: pP_T7_-sfGFP and pP_J23119_-sfGFP, which respectively, express sfGFP from the T7 promoter and from P_J23119_, a strong σ^70^ promoter recognized by the *E. coli* RNA polymerase. On the basis of previous experience and the fact that they have lower total protein concentration (which we assume to roughly correlate with total polymerase and ribosome concentration), we expected the 100 and 25 J lysates (at the same volume) to produce less sfGFP from the same concentration of each plasmid DNA template.

However, protein expression trends were different for the two plasmids. For pP_T7_-sfGFP, the trend was as anticipated: fluorescence decreased with decreasing sonication energy (Figure 1B). However, the 100 and 25 J lysates both yielded higher protein output for pP_J23119_-sfGFP than the 300 J lysate (Figure 1C). Interestingly, the reaction lifetime was longer in these lysates for pP_J23119_-sfGFP, as the fluorescent signal had not yet begun to plateau at 4 h.

While it is not immediately clear why trends in the dependence on sonication energy are different for the two DNA templates, the difference must be due to some *cis* elements on the plasmids because the reactions are otherwise identical. For reporter expression, pP_T7_-sfGFP and pP_J23119_-sfGFP use different RNA polymerases for transcription (T7 vs the native *E. coli* RNA polymerase with the σ^70^ factor) and have different regulatory elements: pP_T7_-sfGFP has the 5' UTR, RBS, and terminator of T7 phage gene 10 while pP_J23119_-sfGFP has a strong *E. coli* RBS and the *rrnB* terminator. However, the vectors also have different origins of replication (ColE1 and p15A, respectively) and selection markers that encode resistance to different antibiotics (kanamycin and chloramphenicol, respectively); these components were not deliberately selected to differ between the two vectors, though their expression levels, while poorly characterized, are likely different and could potentially be related to our findings in Figure 1.

To investigate the causes of the differences in trends, we assembled constructs with different expression regulatory components while maintaining the same backbone (i.e., origin of replication and selection marker of pP_T7_-sfGFP). Since the promoters used in Figure 1 are among the strongest for their transcriptional system but cell-free circuits might require the use of weaker promoters, we tested one additional variant of the T7 promoter (P_T744_) and another σ^70^ promoter (P_J23100_), which are weaker than P_T7_ and P_J23119_. For each promoter, we tested two RBSs (the T7 phage gene 10 RBS and a strong *E. coli* RBS) and two stability hairpins in the 5′ UTR, one derived from the T7 phage gene 10 UTR^14^ and a synthetic hairpin (pHP14^15^).

**Figure 2:**
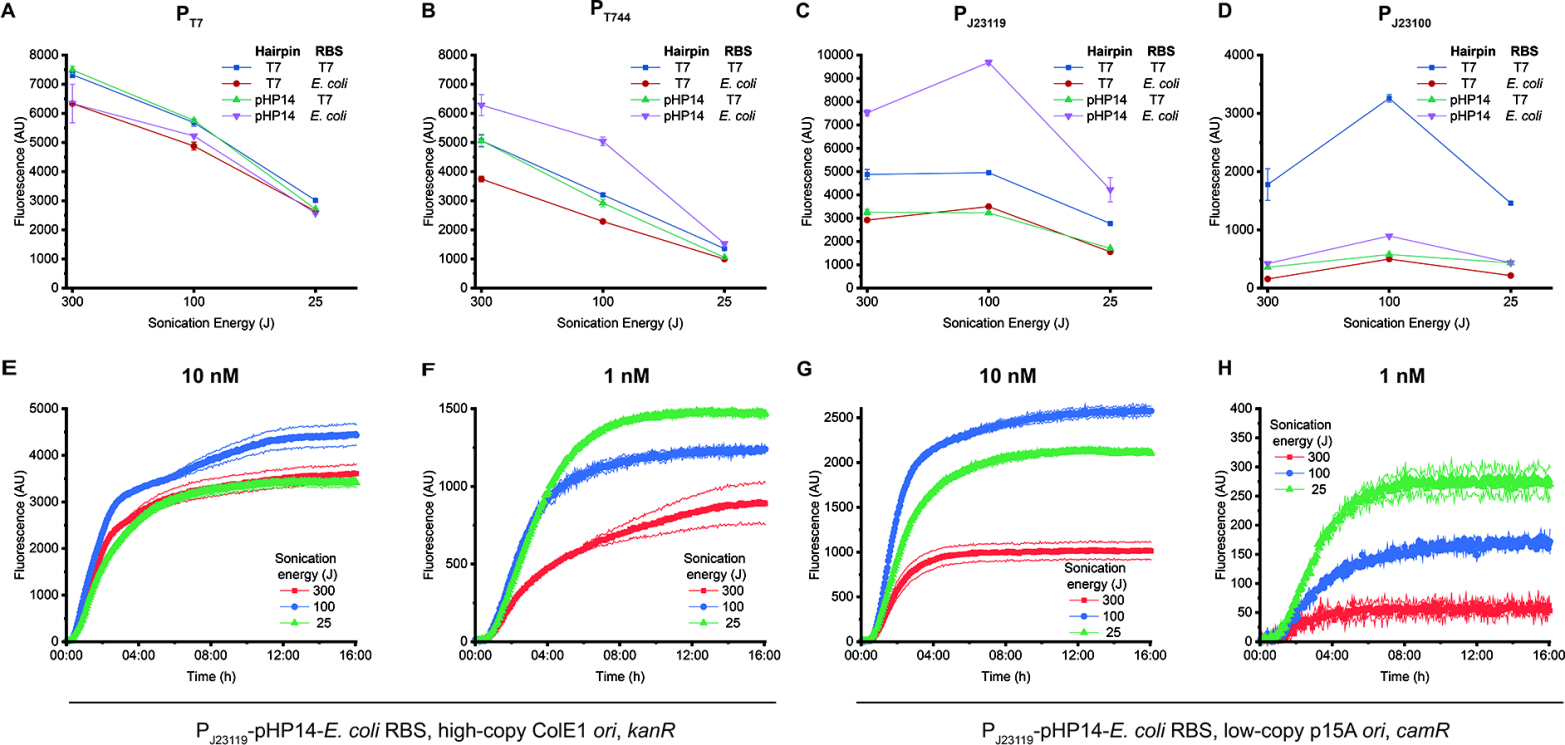
Effects of different *cis* elements and dosage on sfGFP output in lysates prepared using different sonication energy inputs. (A–D) Impact of different RBSs and 5′ UTR hairpins on sfGFP expression from (A) P_T7_, (B) P_T744_, (C) P_J23119_, and (D) P_J23100_. For both T7 promoters, protein output decreases with sonication energy input, and the identities of the hairpin and RBS do not affect the overall trend. For σ^70^ promoters, expression is highest in the 100 J lysate. In panels A–D, plasmid was added at 10 nM and data were collected after 4 h of incubation at 37 °C. (E–H) Impact of plasmid dosage (E vs F, G vs H) and backbone components (E vs G, F vs H). All plasmids contain P_J23119_, the pHP14 hairpin, and the *E. coli* RBS. Plasmid dosage and backbone components both change the trend in sfGFP expression at different sonication energies. In panels E–H, data were collected during incubation at 37 °C. In all panels, error bars indicate the standard deviation of three technical replicates of a representative batch; results for A–D for other batches are shown in Figure S2.

Out of the three components tested, the promoter had the most significant impact on the observed trends. When sfGFP was expressed from T7 promoters, fluorescence always decreased with decreasing sonication energy (Figure 2A,B). The RBS and hairpin affected fluorescence similarly in all lysates. When sfGFP was expressed from σ^70^ promoters, the fluorescent signal was strongest in the 100 J lysate and lowest in the 25 J lysate (Figure 2C,D). The peak at 100 J was most pronounced for pP_J23119_-sfGFP with the pHP14 hairpin and the *E. coli* RBS and for pP_J23100_-sfGFP with the T7 hairpin and RBS. For all other combinations of hairpins and RBSs, the increase at 100 J was not as prominent, though the overall trend remained. These trends were also qualitatively consistent in two other lysate batches prepared over the course of one year (Figure S2), despite expected batch-to-batch variability in the total strength of individual batches. We note that expression being lower at 25 J than at 300 J for the σ^70^ promoters (Figure 2C,D) is not consistent with the data in Figure 1C, suggesting that other elements of the vector (i.e., the origin of replication and the selection marker) may have an impact.

These promoter-driven differences seem to be primarily correlated with the type of promoter (T7 vs σ^70^) rather than its strength. P_J23119_ is a stronger promoter than P_T744_ and yet it produced its highest sfGFP titer in the 100 J lysate. Interestingly, T7 promoters were less robust to changes in sonication energy, with over a 3-fold decrease in fluorescent signal in the 25 J lysate for certain combinations of hairpins and RBSs (Figure 2B). This suggests that T7 RNA polymerase-driven transcription might be primarily limited by the availability of polymerase rather than potentially decreased specific activity of polymerases. On the other hand, transcription using endogenous machinery does not seem to be primarily limited by total availability of polymerase, as it peaks at an intermediate sonication energy. These conclusions may not be completely generalizable, though: for extremely weak promoters P_T773_ and P_T701_ (T7 promoter variants), we observed a trend more consistent with that for σ^70^ promoters (Figure S3), suggesting that promoter strength may still play some role in determining the impact of sonication energy input.

All experiments described so far used a high plasmid concentration (10 nM) at which expression is typically nearly saturated for strong promoters, suggesting it might be valuable to test lower dosages. We tested the construct with P_J23119_, pHP14 hairpin, and *E. coli* RBS at two concentrations: 10 nM (Figure 2E) and 1 nM (Figure 2F). sfGFP expression at 1 nM plasmid was highest in the 25 J lysate, followed by the 100 J lysate. This suggests that in certain concentration regimes, access to machinery may not be the most dominant challenge, giving what is presumably the “quality” of the machinery more impact on expression levels.

Figures 1C and 2E use constructs that differ only in their *ori* and selection marker, yet show quite different relative expression values for 300 J vs 25 J. This surprisingly suggests that parts of the plasmid that are merely artifacts of construct cloning and are typically not considered to have a substantive impact on protein expression can in fact lead to differences in the impact of sonication energy input on expression. We sought to validate this difference in a longer time course using the construct from Figure 1C at 10 nM and 1 nM. At 10 nM, the same trend observed in Figure 1C but not observed in Figure 2E (25 J better than 300 J) remained evident. At 1 nM (Figure 2H), the overall trend was consistent with that observed in Figure 2F, suggesting that in this set of conditions the impact of the vector on sonication-related trends was not substantial. At both plasmid concentrations, sfGFP expression by the low-copy plasmid that confers resistance to chloramphenicol was weaker across all lysates. While these expression vector elements do not play the same role in CFE systems that they do in whole cells, the RNA transcripts and/or proteins they generate in a cell-free reaction may compete for transcriptional and translational resources with potentially significant impact on reporter protein expression.

## Conclusion

The work presented here highlights the substantial impact of cell lysis methods on CFE. Specifically, it shows that our current understanding of the magnitude of this impact (at least for lysis via sonication) is still limited, as previously reported guidelines for determining ideal sonication settings are not generalizable across plasmid components and concentrations. We found that the optimal sonication energy input for a given construct depends on the transcriptional machinery used for the expression product, plasmid dosage, and even plasmid elements not directly involved in reporter expression.

These findings have implications for both the choice of lysis conditions for making crude extracts and the design of genetic circuits for CFE. While we found that high-energy sonication is needed to enable high protein titer from T7 promoters, transcription from native promoters (at least from the promoters tested here) requires more careful selection of the sonication energy, as an intermediate input corresponding to lower total protein content can actually enable stronger plasmid-borne protein expression. Beyond the transcriptional machinery, the origin of replication and the selection marker seem to also have a significant effect on protein output at different sonication energy inputs, perhaps due to their differing RNA and protein expression levels; these components would need more careful effort to select based on their impact on CFE in addition to plasmid cloning requirements.

Our results seem to be correlated with previous studies on crosstalk between genetic cassettes in CFE systems, as genetic cassettes within the plasmids tested in this study seem to affect one another in each of the three lysates to different extents.^16−18^ The degree of cell lysis could potentially have a significant impact on the crosstalk between genetic cassettes, as the sonication energy input affects both the availability and activity of gene expression resources and nucleases. Thus, while using high sonication energy inputs enables thorough cell lysis and leads to greater gene expression machinery concentrations, it can also deactivate this machinery and result in lower activity or higher content of nucleases that will degrade RNA transcripts. There likely is a complex interplay between the quantity and the quality of resources, making it difficult to predict how different genetic cassettes within a plasmid will interact and, ultimately, which sonication energy input will yield the highest protein titers from a given construct.

The extended reaction longevity observed in Figure 1C in extracts prepared using lower sonication energies is another noteworthy result. Increasing the lifetime of cell-free reactions is a key step toward the development of more durable cell-free platforms, but it has not been easily achieved without establishing a continuous^19,20^ or semicontinuous^21^ flux of reagents. Adjusting the degree of cell lysis could be a simple way to extend the longevity of CFE, though this effect does not seem to be generalizable across all constructs tested.

Other variables beyond the sonication energy input, such as the sonication amplitude, affect the degree of cell lysis and CFE and would thus be worth considering in greater depth. Consistent with our findings in Figure 1, changing the amplitude from 50% to 40% or 60% resulted in changes in overall sfGFP output from pP_J23119_-sfGFP that did not directly correlate with the total protein concentration of the extract (Figure S4) but were roughly correlated with the amplitude over that range (Figure S5). Most notably, though, the trends in sfGFP output at different sonication energies were consistent across the different amplitudes, including 100 J of sonication energy being better than both 300 and 25 J for pP_J23119_-sfGFP (Figure S5). These results further highlight the complex impact of different lysis conditions on CFE and suggest that studying other lysis parameters might provide a more complete picture of these effects.

With lysate-based CFE systems becoming an increasingly ubiquitous tool in synthetic biology applications, improving the robustness and reproducibility of these platforms becomes more important. The results reported here show the variation in protein-production capacity that originates from cell lysis, with a complex dependence on changes in the sonication energy input based on multiple confounding factors. Broadly speaking, characterizing the productivity of lysates prepared at different sonication inputs using a single construct at a single set of reaction conditions may be misleading, emphasizing the need for better metrics to characterize cell extracts and the potential need for construct-specific characterization of lysates or more predictive models that can account for many different factors. In addition, there may be more flexibility in the lysis step of the lysate preparation protocol than is often widely thought; the degree of cell lysis can actually be used as an adjustable parameter to improve the extract’s expression potential. Taken together, our results provide valuable insights into the impact of cell lysis, revealing another aspect of CFE systems that needs to be better understood to facilitate their reliable implementation.

## Methods

### Materials

T4 DNA ligase, T5 exonuclease, Taq ligase, Phusion polymerase, Q5 polymerase, and restriction endonucleases were purchased from New England Biolabs (Ipswich, MA, USA). E.Z.N.A. Plasmid Mini and Midi Kits were purchased from Omega Biotek (Norcross, GA, USA), and QIAquick PCR Purification Kits were purchased from QIAGEN (Valencia, CA, USA).

### Strains and Plasmids

*Escherichia coli* K12 DH10B (New England Biolabs, Ipswich, MA) was used for plasmid assembly. *E. coli* BL21 Star (DE3) Δ*lacZ* was used to prepare the extract for cell-free expression. As described above, the plasmids pP_T7_-sfGFP (derived from pJL1, with a ColE1 origin and kanamycin resistance cassette) and pP_J23119_-sfGFP (derived from pJBL7010, with a p15A origin and chloramphenicol resistance cassette) were used as backbone vectors. pP_T7_-sfGFP and pP_J23119_-sfGFP were selected as representative, widely used *E. coli* cell-free vectors spanning the two most common transcription systems. The promoters P_J23119_ and P_J23100_ were obtained from the Anderson promoter collection in the Standard Registry of Biological Parts. The T7 promoter variants were obtained from a study by Komura et al.^22^

### Cloning and Construct Assembly

All constructs were assembled via inverse PCR or Gibson assembly.^23^ LB medium composed of 10 g/L NaCl, 5 g/L yeast extract, and 10 g/L tryptone was used for all cell growth during the cloning steps. Kanamycin (30 μg/mL) and chloramphenicol (30 μg/mL) were used as appropriate for selection. All plasmid sequences are described in the Supporting Information.

### Preparation of Cellular Lysate

Cellular lysate for all experiments was prepared as previously described.^6^ BL21 Star (DE3) Δ*lacZ* cells were grown in 2× YTP medium at 37 °C and 180 rpm to an OD of 1.7, which corresponded with the midexponential growth phase. Cells were then centrifuged at 2700 rcf and washed three times with S30A buffer. S30A buffer contains 50 mM tris, 14 mM magnesium glutamate, 60 mM potassium glutamate, and 2 mM dithiothreitol, and is pH-corrected to 7.7 with acetic acid. After the final centrifugation, the wet cell mass was determined, and cells were resuspended in 1 mL of S30A buffer per 1 g of wet cell mass. The cellular resuspension was divided into 1 mL aliquots. Cells were lysed using a Q125 Sonicator (Qsonica, Newton, CT) with a 3.175 mm diameter probe, at a frequency of 20 kHz, and at 50% of amplitude. Cells were sonicated in 1.5 mL microcentrifuge tubes on ice with cycles of 10 s on, 10 s off, delivering approximately 300, 100, and 25 J (approximately 5 W for each energy input). At the start of each cycle, the tip of the probe was positioned close to the bottom of the tube; approximately 5 times per 10 s cycle, the tube was moved down slowly such that the tip reached the 0.5 mL mark of the tube and then back to its original position. An additional 4 mM of dithiothreitol was added to each tube, and the sonicated mixture was then centrifuged at 12,000 rcf and 4 °C for 10 min. The supernatant was removed, divided into 1 mL aliquots, and incubated at 37 °C and 220 rpm for 80 min. After this runoff reaction, the cellular lysate was centrifuged at 12,000 rcf and 4 °C for 10 min. The supernatant was removed and loaded into a 10 kDa MWCO dialysis cassette (Thermo Fisher). Lysate was dialyzed in 1 L of S30B buffer (14 mM magnesium glutamate, 60 mM potassium glutamate, 1 mM dithiothreitol, pH-corrected to 8.2 with Tris) at 4 °C for 3 h. Dialyzed lysate was removed and centrifuged at 12,000 rcf and 4 °C for 10 min. The supernatant was removed, aliquoted, and stored at −80 °C for future use.

To test the robustness of our results across different lysate batches, we prepared additional batches for each sonication energy input on different days following the protocol described above (Figure S2).

### Cell-Free Reactions

Cell-free reactions for all experiments were run as previously described.^24^ Each cell-free reaction contained 0.85 mM each of GTP, UTP, and CTP, in addition to 1.2 mM ATP, 34 μg/mL of folinic acid, 170 μg/mL *E. coli* tRNA mixture, 130 mM potassium glutamate, 10 mM ammonium glutamate, 12 mM magnesium glutamate, 2 mM each of the 20 standard amino acids, 0.33 mM nicotine adenine dinucleotide (NAD), 0.27 mM coenzyme-A (CoA), 1.5 mM spermidine, 1 mM putrescine, 4 mM sodium oxalate, 33 mM phosphoenol pyruvate (PEP), 27% cell extract, and the specified plasmid concentrations.

Cell-free reactions were run in 10 μL volumes in 384-well small volume plates (Greiner Bio-One), and a clear adhesive film was used to cover the plate and prevent evaporation. Plates were incubated at 37 °C, and fluorescence was measured with a plate reader (Synergy4, BioTek). Excitation and emission for sfGFP were 485 and 510 nm, respectively.

### Bradford Assay

The assay was run as previously described.^4^ In short, a BSA standard curve was prepared at 0, 0.001, 0.002, 0.004, and 0.006 mg/mL in 1 mL cuvettes containing 800 μL of water and 200 μL of Bradford reagent. Two microliters of a 20-fold lysate dilution were added to a cuvette containing the same volumes of water and Bradford reagent. Absorbance was read at 595 nm. Lysate absorbances were compared to the standard curve to determine the total protein concentration.

## Supporting Information Available

(Figures S1–S5) Protein expression at a sonication energy input higher than 300 J, effects of different cis elements on the sfGFP output in multiple lysate batches prepared using different sonication energy inputs, sfGFP expression from weaker T7 promoters, total protein content of extracts as assessed via the Bradford assay, and sfGFP output from pP_J23119_-sfGFP in extracts prepared at different sonication amplitudes and energy inputs; (Tables S1–S2) Annotated sequences of all plasmids and promoter sequences (PDF)
