# Supplementary material for: Complex Dependence of Escherichia coli-based Cell-Free Expression on Sonication Energy During Lysis
Source: ACS Synth Biol. 2023 Sep 19;12(10):3131–6. doi: 10.1021/acssynbio.3c00312 (PMC10594866; doi:10.1021/acssynbio.3c00312)
Supplement: Supplementary file 1 — sb3c00312_si_001.pdf [file sb3c00312_si_001.pdf]

Supporting Information for
Complex Dependence of *Escherichia coli*-based Cell-Free Expression on Sonication Energy During Lysis

Fernanda Piorino and Mark P. Styczynski*

School of Chemical & Biomolecular Engineering, Georgia Institute of Technology, 311 Ferst Drive NW, Atlanta, Georgia 30332, USA

*Email: mark.styczynski@chbe.gatech.edu

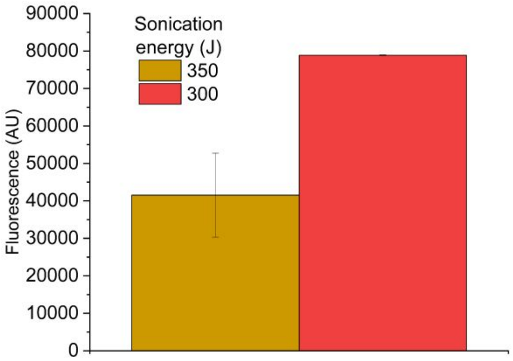

Figure S1. sfGFP expression from pP_{T7}-sfGFP in a lysate prepared at a sonication energy input higher than 300 J. Data were collected after 4 hours of incubation at 37°C; error bars indicate the standard deviation of three technical replicates.

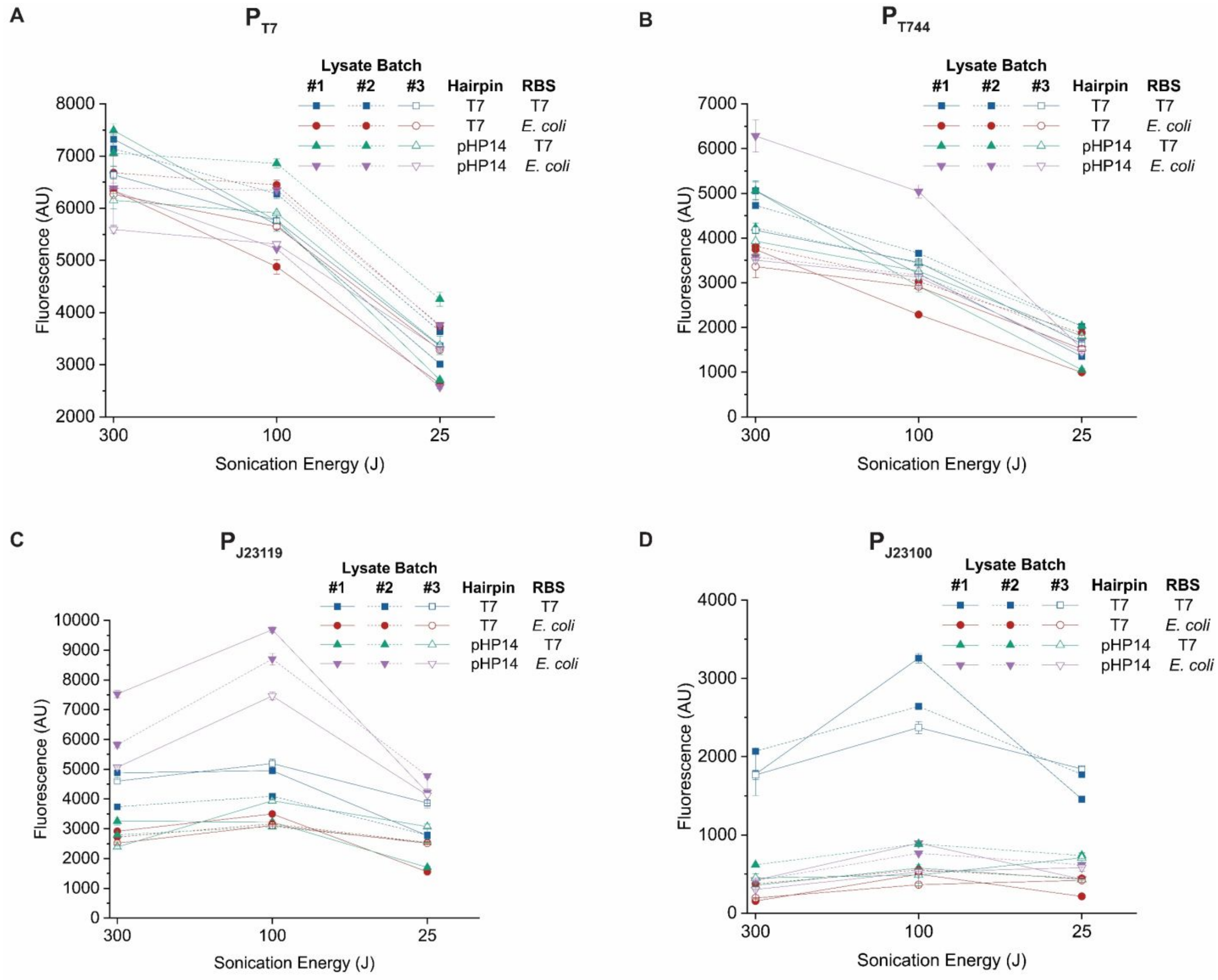

Figure S2. Effects of different *cis* elements on sfGFP output in multiple lysate batches prepared using different sonication energy inputs. For each of the constructs, trends are generally consistent across lysate batches, with some differences in sfGFP output. Data for lysate batch #1 are reproduced from Figure 1. Data were collected after 4 hours of incubation at 37°C; error bars indicate the standard deviation of three technical replicates.

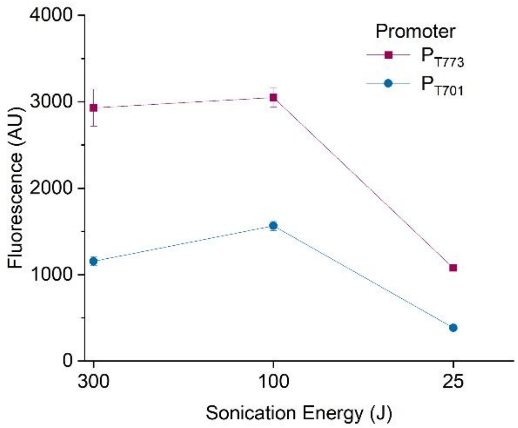

Figure S3. sfGFP expression from weaker T7 promoters, P_{T773} and P_{T701}, in lysates prepared at 300, 100, and 25 J sonication energy inputs. The trend in sfGFP output (strongest expression in the 100 J lysate) is more consistent with the trends observed for σ^{70} promoters. Data were collected after 4 hours of incubation at 37°C; error bars indicate the standard deviation of three technical replicates.

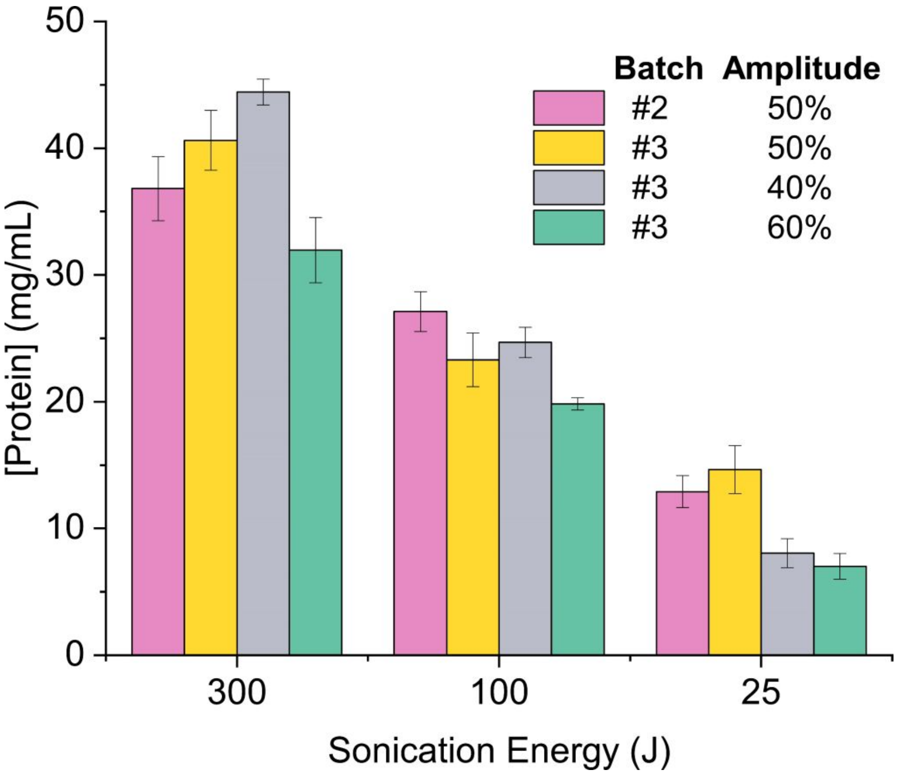

Figure S4. Total protein content of extracts as assessed via Bradford assay. Batches #2 and #3 correspond to the identically named batches in Figure S2. All batch #3 extracts originate from the same cell culture, which was subaliquoted post-harvest and sonicated under different settings. Data were collected after 4 hours of incubation at 37°C; error bars indicate the standard deviation of three technical replicates.

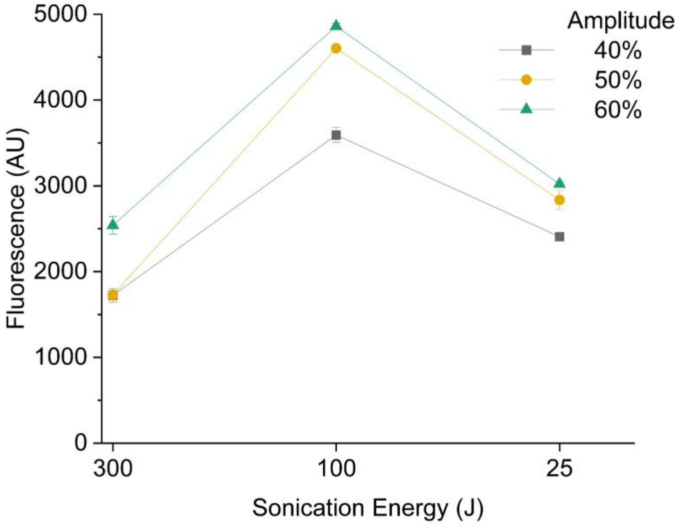

Figure S5. sfGFP output from pP_{J23119}-sfGFP in extracts prepared at different sonication amplitudes and energy inputs. All extracts correspond to lysate batch #3 described in Figure S4. In all reactions, pP_{J23119}-sfGFP was added at 10 nM. Data were collected after 4 hours of incubation at 37°C; error bars indicate the standard deviation of three technical replicates.

Table 1. Annotated sequences of plasmids used in this paper.

<table>
<tr><td>pP_{T7}-sfGFP</td><td>Plasmid encoding sfGFP expression under P_{T7}</td></tr>
<tr><td colspan="2">P_{T7} Stability hairpin RBS sfGFP T7 terminator Kanamycin resistance cassette ColE1 origin
agatcaaaggatcttcttgagatcctttttttctgcgcgtaatctgctgcttgcaaacaaaaaaaccaccgctaccagcggtggtttgtttgcc
ggatcaagagctaccaactctttttccgaaggtaactggcttcagcagagcgcagataccaaatactgttcttctagtgtagccgtagttag
gccaccacttcaagaactctgtagcaccgcctacatacctcgctctgctaatcctgttaccagtggctgctgccagtggcgataagtcgtg
tcttaccgggttggactcaagacgatagttaccggataaggcgcagcggtcgggctgaacggggggttcgtgcacacagcccagcttg
gagcgaacgacctacaccgaactgagatacctacagcgtgagctatgagaaagcgccacgcttcccgaagggagaaaggcggaca
ggtatccggtaagcggcagggtcggaacaggagagcgcacgagggagcttccagggggaaacgcctggtatctttatagtcctgtcg
ggtttcgccacctctgacttgagcgtcgatttttgtgatgctcgtcaggggggcggagcctatggaaaaacgccagcaacgcgatcccg
cgaaattaatacgactcactatagggagaccacaacggtttccctctagaaataattttgtttaactttaagaaggagatatacatATGA
GCAAAGGTGAAGAACTGTTTACCGGCGTTGTGCCGATTCTGGTGGAACTGGATGG
CGATGTGAACGGTCACAAATTCAGCGTGCGTGGTGAAGGTGAAGGCGATGCCACG
ATTGGCAAACTGACGCTGAAATTTATCTGCACCACCGGCAAACTGCCGGTGCCGT
GGCCGACGCTGGTGACCACCCTGACCTATGGCGTTCAGTGTTTTAGTCGCTATCCG
GATCACATGAAACGTCACGATTTCTTTAAATCTGCAATGCCGGAAGGCTATGTGC
AGGAACGTACGATTAGCTTTAAAGATGATGGCAAATATAAAACGCGCGCCGTTGT
GAAATTTGAAGGCGATACCCTGGTGAACCGCATTGAACTGAAAGGCACGGATTTT
AAAGAAGATGGCAATATCCTGGGCCATAAACTGGAATACAACTTTAATAGCCATA
ATGTTTATATTACGGCGGATAAACAGAAAAATGGCATCAAAGCGAATTTTACCGT
TCGCCATAACGTTGAAGATGGCAGTGTGCAGCTGGCAGATCATTATCAGCAGAAT
ACCCCGATTGGTGATGGTCCGGTGCTGCTGCCGGATAATCATTATCTGAGCACGCA
GACCGTTCTGTCTAAAGATCCGAACGAAAAAGGCACGCGGGACCACATGGTTCTG
CACGAATATGTGAATGCGGCAGGTATTACGTGGAGCCATCCGCAGTTCGAAAAAT</td></tr>
</table>

AAgtcgaccggctgctaacaaagcccgaaaggaagctgagttggctgctgccaccgctgagcaataactagcataaccccttgggg
cctctaaacgggtcttgaggggttttttgctgaaagccaattctgattagaaaaactcatcgagcatcaaatgaaactgcaatttattcatatc
aggattatcaataccatatttttgaaaaagccgtttctgtaatgaaggagaaaactcaccgaggcagttccataggatggcaagatcctggt
atcggtctgcgattccgactcgtccaacatcaatacaacctattaatttcccctcgtcaaaaataaggttatcaagtgagaaatcaccatga
gtgacgactgaatccggtgagaatggcaaaagcttatgcatttctttccagacttgttcaacaggccagccattacgctcgtcatcaaaatc
actcgcatcaaccaaaccgttattcattcgtgattgcgcctgagcgagacgaaatacgcgatcgctgttaaaaggacaattacaaacagg
aatcgaatgcaaccggcgcaggaacactgccagcgcatcaacaatattttcacctgaatcaggatattcttctaatacctggaatgctgttt
tcccggggatcgcagtggtgagtaaccatgcatcatcaggagtacggataaaatgcttgatggtcggaagaggcataaattccgtcagc
cagtttagtctgaccatctcatctgtaacatcattggcaacgctacctttgccatgtttcagaaacaactctggcgcatcgggcttcccatac
aatcgatagattgtcgcacctgattgcccgacattatcgcgagcccatttatacccatataaatcagcatccatgttggaatttaatcgcggc
ttcgagcaagacgtttcccgttgaatatggctcataacacccttgtattactgtttatgtaagcagacagttttattgttcatgatgatatattttt
atcttgtgcaatgtaacatcagagattttgagacacaacgtg

<table>
<tr><td>pP$_{J23119}$-sfGFP</td><td>Plasmid encoding sfGFP expression under P$_{J23119}$</td></tr>
<tr><td colspan="2">P$_{J23119}$ pHP14 hairpin RBS sfGFP TrrnB terminator Chloramphenicol resistance cassette p15A origin

ttgacagctagctcagtcctaggtataatactagtACGTCGACTCTCGAGTGAGATTGTTGACGGTACCG
TATTTTggatctaggaggaaggatctatgagcaaaggagaagaacttttcactggagttgtcccaattcttgttgaattagatggtgat
gttaatgggcacaaattttctgtccgtggagagggtgaaggtgatgctacaaacggaaaactcacccttaaatttatttgcactactggaaa
actacctgttccgtggccaacacttgtcactactctgacctatggtgttcaatgcttttcccgttatccggatcacatgaaacggcatgactttt
tcaagagtgccatgcccgaaggttatgtacaggaacgcactatatctttcaaagatgacgggacctacaagacgcgtgctgaagtcaag
tttgaaggtgatacccttgttaatcgtatcgagttaaagggtattgattttaaagaagatggaaacattcttggacacaaactcgagtacaact
ttaactcacacaatgtatacatcacggcagacaaacaaaagaatggaatcaaagctaacttcaaaattcgccacaacgttgaagatggttc
cgttcaactagcagaccattatcaacaaaatactccaattggcgatggccctgtccttttaccagacaaccattacctgtcgacacaatctgt
cctttcgaaagatcccaacgaaaagcgtgaccacatggtccttcttgagtttgtaactgctgctgggattacacatggcatggatgagctct
acaaataaggatctgaagcttgggcccgaacaaaaactcatctcagaagaggatctgaatagcgccgtcgaccatcatcatcatcatcat
tgagtttaaacggtctccagcttggctgttttggcggatgagagaagattttcagcctgatacagattaaatcagaacgcagaagcggtct
gataaaacagaatttgcctggcggcagtagcgcggtggtcccacctgaccccatgccgaactcagaagtgaaacgccgtagcgccga
tggtagtgtggggtctccccatgcgagagtagggaactgccaggcatcaaataaaacgaaaggctcagtcgaaagactgggcctttcg
ttttatctgttgtttgtcggtgaactggatccttactcgagtctagactgcagttgatcgggcacgtaagaggttccaactttcaccataatga
aataagatcactaccgggcgtattttttgagttatcgagattttcaggagctaaggaagctaaaatggagaaaaaaatcactggatatacca
ccgttgatatatcccaatggcatcgtaaagaacattttgaggcatttcagtcagttgctcaatgtacctataaccagaccgttcagctggata
ttacggcctttttaaagaccgtaaagaaaaataagcacaagttttatccggcctttattcacattcttgcccgcctgatgaatgctcatccgga
atttcgtatggcaatgaaagacggtgagctggtgatatgggatagtgttcacccttgttacaccgttttccatgagcaaactgaaacgttttc
atcgctctggagtgaataccacgacgatttccggcagtttctacacatatattcgcaagatgtggcgtgttacggtgaaaacctggcctattt
ccctaaagggtttattgagaatatgtttttcgtctcagccaatccctgggtgagtttcaccagttttgatttaaacgtggccaatatggacaact
tcttcgcccccgttttcaccatgggcaaatattatacgcaaggcgacaaggtgctgatgccgctggcgattcaggttcatcatgccgtttgt
gatggcttccatgtcggcagaatgcttaatgaattacaacagtactgcgatgagtggcagggcggggcgtaatttgatatcgagctcgctt
ggactcctgttgatagatccagtaatgacctcagaactccatctggatttgttcagaacgctcggttgccgccgggcgttttttattggtgag
aatccaagcctccgatcaacgtctcattttcgccaaaagttggcccagggcttcccggtatcaacagggacaccaggatttatttattctgc
gaagtgatcttccgtcacaggtatttattcggcgcaaagtgcgtcgggtgatgctgccaacttactgatttagtgtatgatggtgtttttgagg
tgctccagtggcttctgtttctatcagctgtccctcctgttcagctactgacggggtggtgcgtaacggcaaaagcaccgccggacaAAt
cagcgctagcggagtgtatactggcttactatgttggcactgatgagggtgtcagtgaagtgcttcatgtggcaggagaaaaaaggctgc
accggtgcgtcagcagaatatgtgatacaggatatattccgcttcctcgctcactgactcgctacgctcggtcgttcgactgcggcgagc
ggaaatggcttacgaacggggcggagatttcctggaagatgccaggaagatacttaacagggaagtgagagggccgcggcaaagcc</td></tr>
</table>

gtttttccataggctccgcccccctgacaagcatcacgaaatctgacgctcaaatcagtggtggcgaaacccgacaggactataaagata
ccaggcgtttccccctggcggctccctcgtgcgctctcctgttcctgcctttcggtttaccggtgtcattccgctgttatggccgcgtttgtct
cattccacgcctgacactcagttccgggtaggcagttcgctccaagctggactgtatgcacgaaccccccgttcagtccgaccgctgcg
ccttatccggtaactatcgtcttgagtccaacccggaaagacatgcaaaagcaccactggcagcagccactggtaattgatttagaggag
ttagtcttgaagtcatgcgccggttaaggctaaactgaaaggacaagttttggtgactgcgctcctccaagccagttacctcggttcaaag
agttggtagctcagagaaccttcgaaaaaccgccctgcaaggcggttttttcgttttcagagcaagagattacgcgcagaccaaaacgat
ctcaagaagatcatcttattaatcagataaaatatttctagatttcagtgcaatttatctcttcaaatgtagcacctgaagtcagccccatacga
tataagttgtaattctcatgtttgacagcttatcatcgataagcttccgatggcgcgccgagaggctttacactttatgcttccggctgaattct
aaagatct

Table 2. Sequences of all promoters tested.

Promoter	Sequence
P_{T7}	taatacgactcactatagggaga
P_{T773}	taatacgactcactaaagggaga
P_{T744}	atatacgactcactatagggaga
P_{T701}	aaatgagactcactacagggaga
P_{J23119}	ttgacagctagctcagtcctaggtataatactagt
P_{J23100}	ttgacggctagctcagtcctaggtacagtgctagc
